# Predicting of axillary lymph node metastasis in invasive breast cancer using multiparametric MRI dataset based on CNN model

**DOI:** 10.3389/fonc.2022.1069733

**Published:** 2022-12-06

**Authors:** Xiaodong Zhang, Menghan Liu, Wanqing Ren, Jingxiang Sun, Kesong Wang, Xiaoming Xi, Guang Zhang

**Affiliations:** ^1^ Department of Radiology, The First Affiliated Hospital of Shandong First Medical University, Jinan, China; ^2^ Postgraduate Department, Shandong First Medical University (Shandong Academy of Medical Sciences), Jinan, China; ^3^ Department of Health Management, The First Affiliated Hospital of Shandong First Medical University, Jinan, China; ^4^ School of Computer Science and Technology, Shandong Jianzhu University, Jinan, China

**Keywords:** breast cancer, magnetic resonance imaging, axillary lymph node, metastasis, convolutional neural network

## Abstract

**Purpose:**

To develop a multiparametric MRI model for predicting axillary lymph node metastasis in invasive breast cancer.

**Methods:**

Clinical data and T2WI, DWI, and DCE-MRI images of 252 patients with invasive breast cancer were retrospectively analyzed and divided into the axillary lymph node metastasis (ALNM) group and non-ALNM group using biopsy results as a reference standard. The regions of interest (ROI) in T2WI, DWI, and DCE-MRI images were segmented using MATLAB software, and the ROI was unified into 224 × 224 sizes, followed by image normalization as input to T2WI, DWI, and DCE-MRI models, all of which were based on ResNet 50 networks. The idea of a weighted voting method in ensemble learning was employed, and then T2WI, DWI, and DCE-MRI models were used as the base models to construct a multiparametric MRI model. The entire dataset was randomly divided into training sets and testing sets (the training set 202 cases, including 78 ALNM, 124 non-ALNM; the testing set 50 cases, including 20 ALNM, 30 non-ALNM). Accuracy, sensitivity, specificity, positive predictive value, and negative predictive value of models were calculated. The receiver operating characteristic (ROC) curve and area under the curve (AUC) were used to evaluate the diagnostic performance of each model for axillary lymph node metastasis, and the DeLong test was performed, P< 0.05 statistically significant.

**Results:**

For the assessment of axillary lymph node status in invasive breast cancer on the test set, multiparametric MRI models yielded an AUC of 0.913 (95% CI, 0.799-0.974); T2WI-based model yielded an AUC of 0.908 (95% CI, 0.792-0.971); DWI-based model achieved an AUC of 0.702 (95% CI, 0.556-0.823); and the AUC of the DCE-MRI-based model was 0.572 (95% CI, 0.424-0.711). The improvement in the diagnostic performance of the multiparametric MRI model compared with the DWI and DCE-MRI-based models were significant (P< 0.01 for both). However, the increase was not meaningful compared with the T2WI-based model (P = 0.917).

**Conclusion:**

Multiparametric MRI image analysis based on an ensemble CNN model with deep learning is of practical application and extension for preoperative prediction of axillary lymph node metastasis in invasive breast cancer.

## Introduction

Global cancer statistics for 2020 show that breast cancer has surpassed lung cancer as the most common cancer in women and the leading cause of cancer deaths in women (1). Lymphatic metastasis is the most common route for breast cancer, and the status of axillary lymph nodes becomes an important indicator to determine the clinical stage, treatment method, and prognosis (2, 3). Axillary lymph node dissection (ALND) and sentinel lymph node biopsy (SLNB) are the clinical standards for the evaluation of axillary lymph node metastasis (ALNM). Since postoperative complications after ALND are challenging to recover from, such as narrow shoulder and upper arm movement, lymphedema, Etc., ALND is now gradually being replaced by SLNB (4–7). Although SLNB has become the primary means of evaluating the status of axillary lymph nodes, the negative rate of SLNB reaches 70% (8), and the incidence of postoperative complications of SLNB reaches 41% (7), which still has a significant impact on the prognosis of breast cancer patients. Therefore, preoperative non-invasive and accurate axillary lymph node status evaluation is of clinical importance.

Currently, the main non-invasive methods for preoperative assessment of axillary lymph node status are imaging examinations, including mammography, ultrasound, magnetic resonance imaging (MRI), and PET/CT (9–13). However, mammography cannot show the entire axilla, and only the anterior and inferior portions of the axilla can be observed. Ultrasound is not only limited to superficial lymph nodes, but is also determined by the diagnostic experience of the doctor. PET/CT is expensive and may expose the patient to ionizing radiation. Nevertheless, MRI has excellent tissue resolution and is the best imaging examination to evaluate breast cancer and axillary lymph nodes (14, 15). Radiologists usually observe axillary lymph node morphology, size, fat hilum, and cortical thickness in MR images to diagnose the occurrence of metastasis (16, 17). However, when axillary lymph nodes are small and have poor contrast with surrounding tissues, radiologists have difficulty interpreting them. As a result, the sensitivity of conventional breast MRI for detecting axillary lymph node metastasis is still moderate (18). Therefore, it cannot be a definitive evaluation method for axillary lymph node metastasis. In addition, previous studies have shown an intrinsic connection between preoperative MRI images of the breast cancer tumor and axillary lymph node metastasis, including anatomical and functional sequences (19–22). However, this inherent connection is difficult for radiologists to observe.

In the past few years, deep learning methods have been increasingly used for medical image processing and are evolving into a new field of medical imaging. Deep learning (23) differs from radiomics, an end-to-end learning model that automatically learns and extracts the intrinsic features of medical images and then completes the corresponding tasks, avoiding the human factor and omitting the traditional complex operational steps. The deep learning using the MRI dataset applied to axillary lymph node metastasis in breast cancer mainly focuses on axillary lymph nodes and breast cancer tumors. Convolutional neural networks (CNN) are commonly used as a conventional deep learning model in breast image analysis. Ha, et al. (24) and Ren et al. (25) used MR images of axillary lymph nodes to build CNN models for their studies, and the studies by Luo et al. (26) and Wang et al. (27)used anatomical and functional MR images of breast cancer tumors. All of their deep-learning models achieved good prediction results.

This study aims to develop an innovative prediction model that can differentiate between ALNM and non-ALNM in breast cancer using multiparametric MRI datasets based on the CNN model.

## Materials and methods

### Population

**Figure 1:**
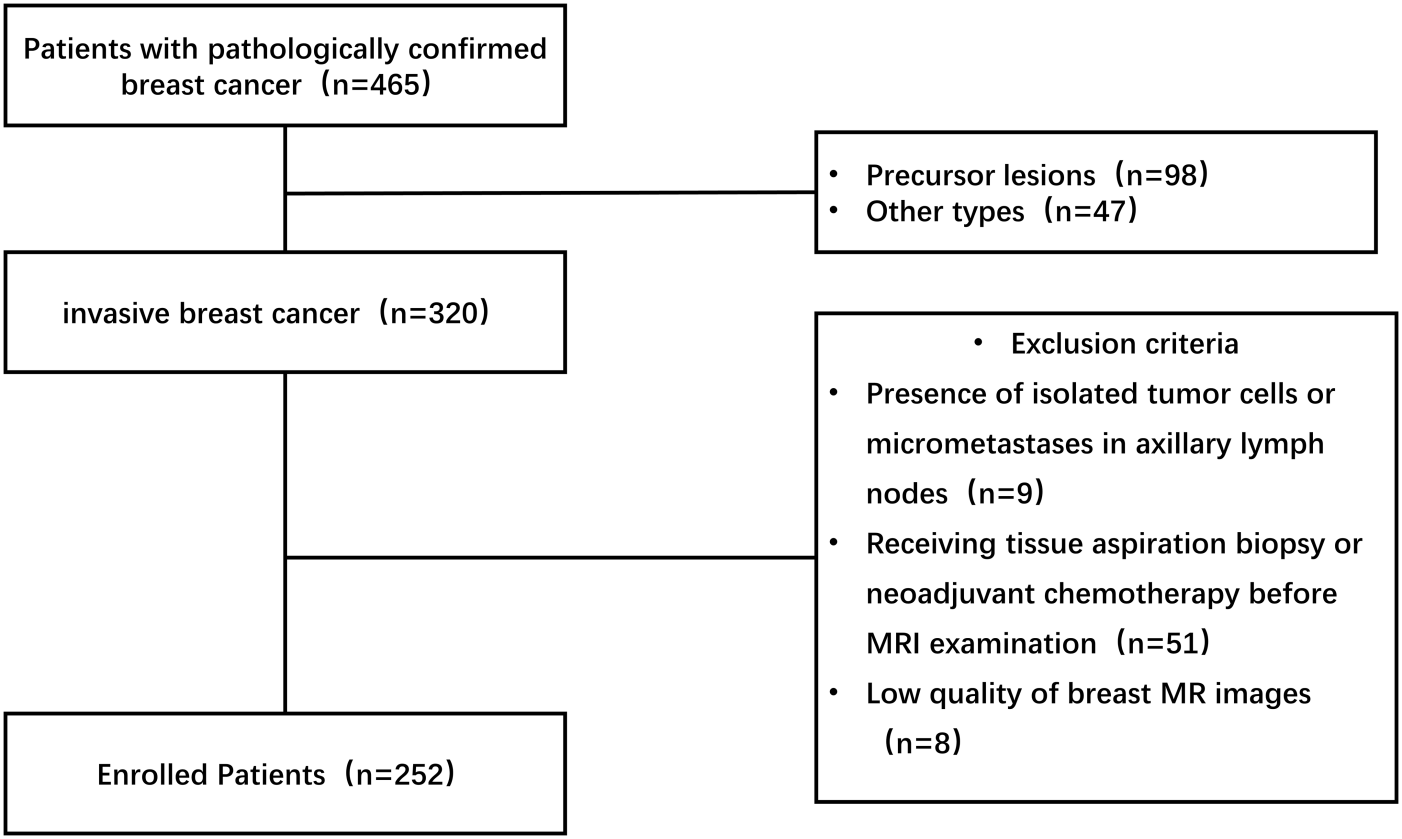
Process of enrolling patients with invasive breast cancer.

We retrospectively studied 465 breast cancer patients from January 2016 to June 2022. The study was approved by the ethics committee and institutional review and waived the requirement for informed consent. Inclusion criteria for this study: (1) invasive breast cancer confirmed by surgical pathology and axillary lymph node status also confirmed by postoperative pathology; (2) pathological stage ≥ pN1a was selected for the ALNM group and pN0 for the non-ALNM group; (3) all preoperative breast MRI scan and enhancement examinations were done. Exclusion criteria: (1) pathological findings showing the presence of isolated tumor cells in axillary lymph nodes or the occurrence of micrometastases; (2) receiving tissue aspiration biopsy or neoadjuvant chemotherapy before MRI examination; (3) the presence of artifacts in MRI images. The specific inclusion and exclusion process is illustrated in **Figure 1**. 252 patients with invasive breast cancer, including 98 with ALNM and 154 with non-ALNM, were finally included in our study, and the clinical characteristics of these people are shown in **Table 1**.

**Table 1:** Population characteristics.

	Characteristics	Total	ALNM	Non-ALNM	P
	number	n=252	n=98	n=154	
All patients	Age (mean ± SD), years old	51.3 ± 11.24	51.0 ± 11.05	51.6 ± 11.40	0.698
	Tumor size (mean ± SD), cm	2.1 ± 0.97	2.4 ± 1.15	1.9 ± 0.78	<0.01
	Tumor margin				0.079
	Regular	102 (40.5)	33 (33.7)	69 (44.8))	
	Irregular	150 (59.5)	65 (66.3)	85 (55.2)	
	TIC curve type				0.201
	II	168 (66.7)	70 (71.4)	98 (63.6)	
	III	84 (33.3)	28 (28.6)	56 (36.4)	
	Number	n=50	n=20	n=30	
Testing dataset	Age (mean ± SD), years old	50.7 ± 10.74	49.1 ± 11.77	51.7 ± 10.06	0.394
	Tumor size (mean ± SD), cm	2.0 ± 0.80	2.3 ± 0.92	1.9 ± 0.67	0.086
	Tumor margin				0.387
	Regular	22 (44.0)	7 (35.0)	15 (50.0)	
	Irregular	28 (56.0)	13 (65.0)	15 (50.0)	
	TIC curve type				0.243
	II	29 (58.0)	14 (70.0)	15 (50.0)	
	III	21 (42.0)	6 (30.0)	15 (50.0)	

ALNM, axillary lymph node metastasis; SD, standard deviation; TIC, time-intensity curve.

### MR imaging parameters

Breast MRI was performed using a 3.0T MRI scanner (Magnetom Skyra, Siemens, Germany) and a 1.5T MRI scanner (Signa Explorer, GE, USA) with dedicated breast coils. Both scanners acquired the following sequences: axial T2-weighted imaging (T2WI), axial diffusion-weighted imaging (DWI), and axial dynamic contrast-enhanced magnetic resonance imaging (DCE-MRI) images, and DCE-MRI sequences were imaged by injecting 15 ml of gadolinium contrast medium (Magnevist, Bayer Schering, Germany) at a rate of 3.0 ml/s, and a total of 7 phases were performed. Only the phase with the most significant enhancement, determined by the time-intensity curve (TIC), was used in the DCE-MRI images. The specific parameters are shown in **Table 2**.

**Table 2:** Magnetic Resonance Imaging Scanner Parameters.

MRI parameters	1.5T			3.0T		
	T2WI	DWI	DCE-MRI	T2WI	DWI	DCE-MRI
TR (ms)	7210	5030	5.6	4300	6650	3.6
TE (ms)	102	56	1.7	75	65	1.8
Flip angle	160°	180°	15°	120°	180°	10°
Slice gap (mm)	1	1	0.5	0.8	1.1	0.5
Slice thickness(mm)	4	5	1.5	4	4	2
FOV (mm²)	320×320	320×320	320×320	351×351	340×340	340×340
Matrix	288×224	110×170	114×224	576×576	224×224	512×512
b value (s/mm²)	—	50,800	—	—	50,1000	—

TR, repetition time; TE, echo time; FOV, the field of view; T2WI, T2-weighted imaging; DWI, diffusion weighting imaging; DCE-MRI, dynamic contrast-enhanced magnetic resonance imaging.

### Image processing and base model building

**Figure 2:**
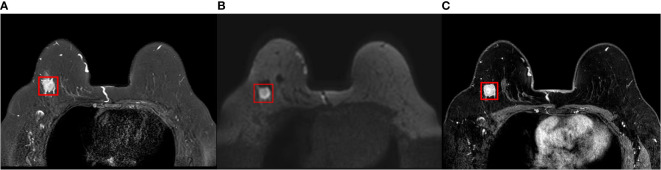
Cropping of ROI in raw MRI images of invasive breast cancer. **(A)** T2WI of the breast; **(B)** DWI of the breast; **(C)** Period with the most significant enhancement in DCE-MRI of the breast. ROI, region of interest; T2WI, T2-weighted imaging; DWI, diffusion weighting imaging; DCE-MRI, dynamic contrast-enhanced magnetic resonance imaging.

The clipping of the lesion region of interest (ROI) included the breast tumor and peritumoral area in this study, not the axillary lymph nodes. Two doctors with three years of breast imaging experience determined the tumor’s location, size, and borders. When a dispute arose, a senior doctor with fifteen years of experience decided it independently. We later used Matlab-R2018b (Math works, Massachusetts, USA) software to crop out the ROI from T2WI, DWI, and DCE-MRI raw images of 252 breast cancer, and the ROI segmentation example is in **Figure 2**. First, boundary boxes were formed around each axial 2D ROI for layer-by-layer ROI segmentation. Then, all segmented 2D ROI images were unified into 224×224. Finally, image normalization was performed by equation (1) so that the pixel values fall in the [0,1] interval.


(1) $$Norm=\frac{xi-min\left(x\right)}{max\left(x\right)-min\left(x\right)}$$

Where *xi* represents the image pixel value, *max*(*x*), *min*(*x*) represent the maximum and minimum values of the image pixels, respectively. All datasets were randomly divided into training and test sets, with 202 cases in the training set, including 78 cases of ALNM and 124 cases of non-ALNM; 50 cases in the test set, including 20 cases of ALNM and 30 cases of non-ALNM.

**Figure 3:**
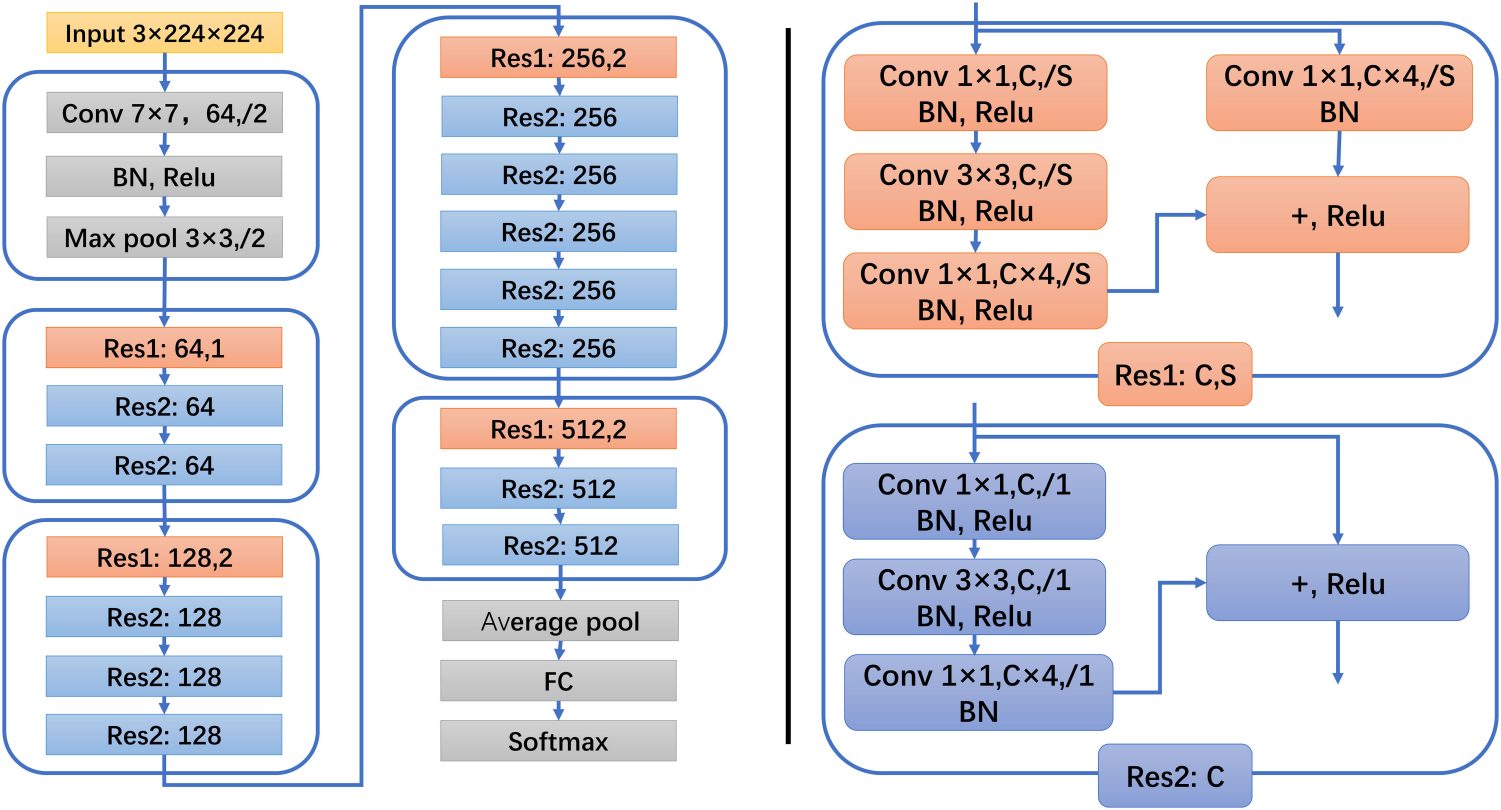
ResNet50 architecture. Res1 and Res2 represent two kinds of residual block structures, C represents the number of convolution kernels, and S represents the step distance.

For the outstanding image feature extraction ability and classification accuracy of residual network (ResNet), the base model in this study is constructed based on ResNet50. The ResNet50 architecture is shown in **Figure 3**. In this study, T2WI, DWI, and DCE-MRI of breast cancer tumors were used as input, and the feature classifier Softmax score threshold 0.5 was used for classification. Three base models of T2WI, DWI, and DCE-MRI were constructed to distinguish between ALNM and non-ALNM. ResNet50 models were trained and tested on a Windows image workstation using Python, the open-source deep learning library torch, and math architecture with an NVIDIA GeForce GTX 2080ti GPU graphics processor. In our study, we used the training set data for training and fine-tuning the parameters of ResNet50, initially we set the learning rate interval to 0.001-0.000001 and the batch size to 16, by gradually adjusting the batch size (16-32-64) and learning rate to make ResNet50 more suitable for our research task. Finally, during the training process, we determined the learning rate be 0.00005, the batch size to be 64, and the number of training epochs was set to 300.

### Multiparametric MRI combined with an ensemble learning model

**Figure 4:**
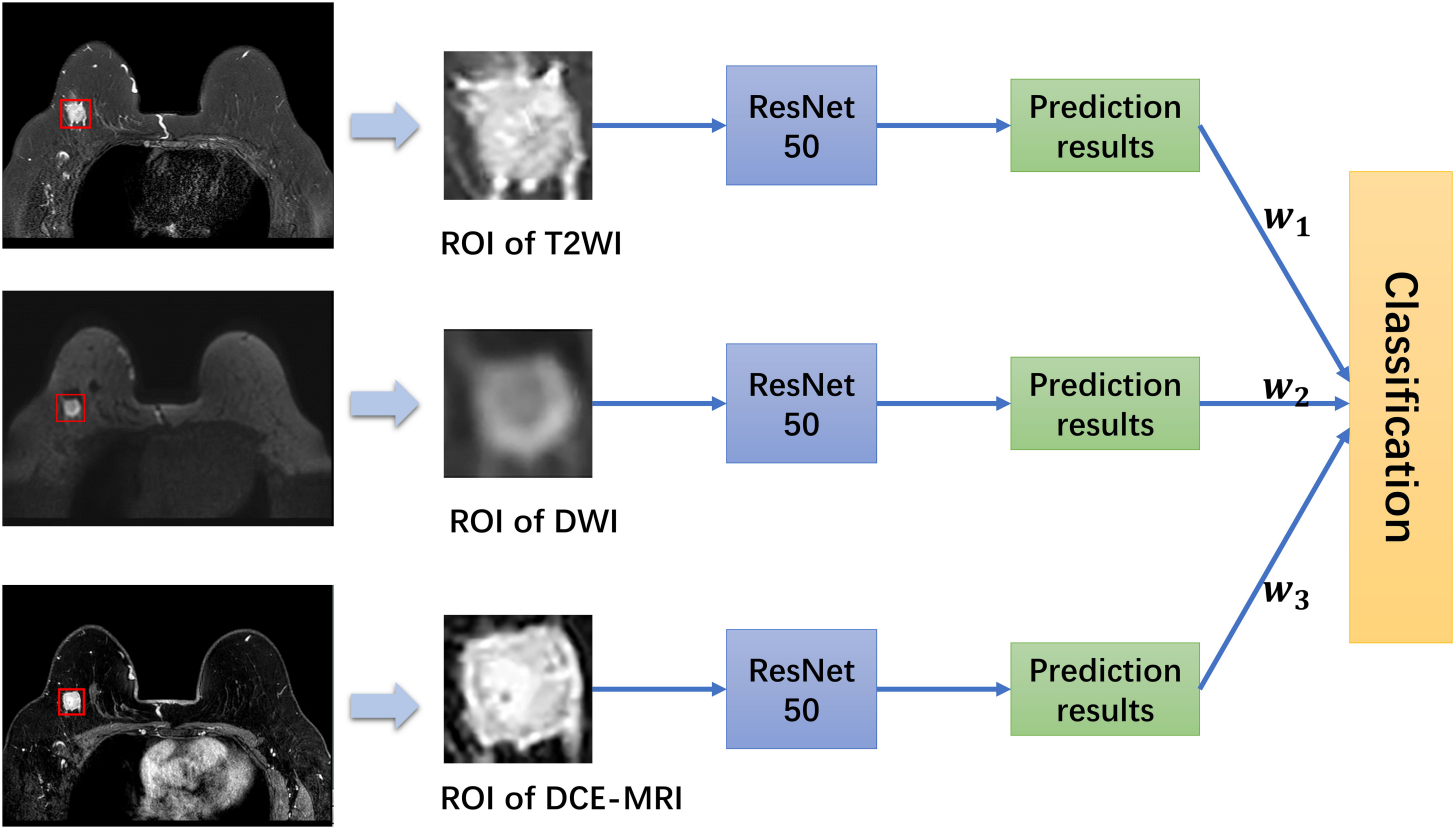
Workflow and the whole structure of the multiparametric MRI model. w represents the weight of each base model.

Ensemble learning is a machine learning model that accomplishes the learning task by constructing and combining multiple base models so that multiple weak classification models can be turned into a single robust model (28). In this study, the idea of a weighted voting method in ensemble learning was adopted, and three base models, T2WI, DWI, and DCE-MRI, were applied as part of the novel prediction model we studied. The workflow of the integrated model is shown in **Figure 4**.

In this work, the trained three base models of T2WI, DWI, and DCE-MRI were tested, the weight value of each base model was calculated according to equation (2), and then a comprehensive decision was made by equation (3). Finally, half of the sum of the weights of the three base models were used as the threshold to make the final classification.


(2) $$w_{i}=\frac{\sum_{i=1}^{3}ACC_{i}}{3}$$

Where *ACC_i_* represents the accuracy of each base model on the test set, and *w_i_* refers to the classification weight of each base model.


(3) $$H\left(x\right)=\sum_{i=1}^{3}w_{i}h_{i}\left(x\right)$$

Where *h_i_* (*x*) represents the probability value of the output of each base model for the same case in the test set, and *H*(*x*) refers to the final probability value of the output of the multi-parameter combined integrated learning model. The multiparametric MRI model combined the information from the three base models of T2WI, DWI, and DCE-MRI to predict the status of axillary lymph nodes, which was more consistent with the clinical diagnostic environment.

### Statistical analysis

SPSS 22.0 and MedCalc 20.0 were used for statistical analysis, the Kolmogorov–Smirnov test was used for normality, and quantitative data conforming to a normal distribution were expressed using the mean ± standard deviation. Independent samples t-test was used for continuous data between ALNM and Non-ALNM groups, and χ² test or Fisher’s exact test was used for categorical data. P< 0.05 was statistically significant. Model assessment indexes were used to evaluate the diagnostic performance of the base models and multiparametric model for axillary lymph node metastasis, including accuracy, sensitivity, specificity, positive predictive value, negative predictive value, receiver operating characteristic (ROC) curve, and area under the curve (AUC). The DeLong test evaluated the ROC curves between different models, and P< 0.05 was considered statistically significant.

## Results

The mean age of all patients was 51.3 ± 11.24 years old, the mean age of the ALNM group was 51.0 ± 11.05 years old, and the mean age of the non-ALNM group was 51.6 ± 11.40 years old. There was no significant difference in age, tumor margin, and TIC curve type between the two groups of ALNM and non-ALNM, except for tumor size. This statistic is consistent with previous studies (29, 30) but not affecting our deep-learning experiment. In the testing set, the mean age of the ALNM group was 49.1 ± 11.77 years, and the mean tumor size was 2.3 ± 0.92 cm, the mean age of the non-ALNM group was 51.7 ± 10.06 years, and the mean tumor size was 1.9 ± 0.67cm. There were no significant differences between the ALNM and non-ALNM groups regarding age, tumor size, tumor margin, or the type of TIC curve.

**Figure 5:**
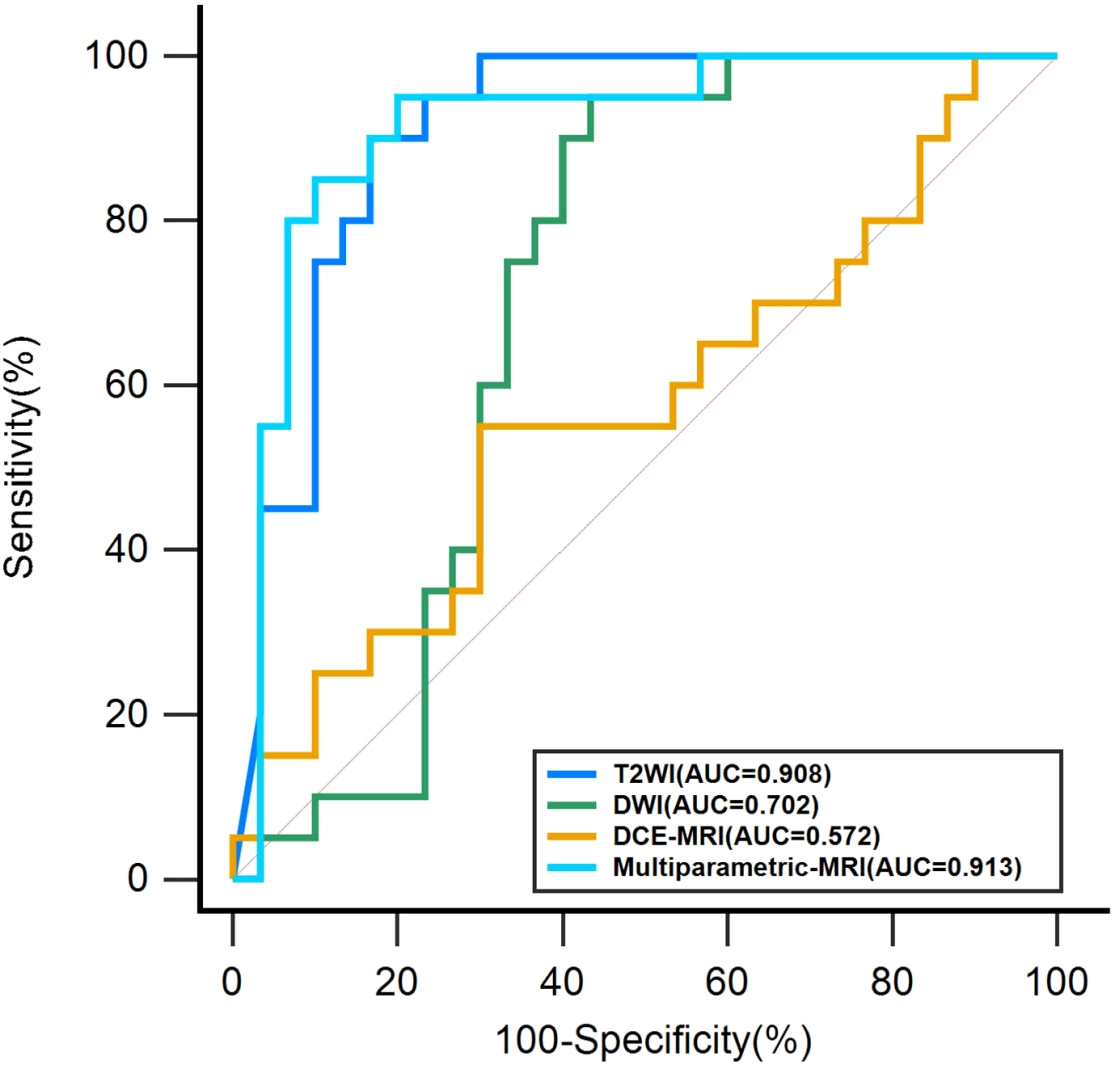
The Roc curves of the base and multiparametric MRI models.

The model indicators are presented in **Table 3**. The Roc curves of the base and multiparametric MRI models are shown in **Figure 5**. The AUC of the T2WI-based model was 0.908 (95% CI, 0.792-0.971), with an accuracy of 0.840, a sensitivity of 0.750, a specificity of 0.900, a PPV of 0.833, and an NPV of 0.844. The AUC of the DWI-based model was 0.702 (95% CI, 0.556-0.823), with an accuracy of 0.640, a sensitivity of 0.550, a specificity of 0.700, a PPV of 0.550, and an NPV of 0.700. The AUC of the DCE-MRI-based model was 0.572 (95% CI, 0.424-0.711), with an accuracy of 0.540, a sensitivity of 0.550, a specificity of 0.533, a PPV of 0.440, and an NPV of 0.640. The AUC of the multiparametric MRI model was 0.913 (95% CI, 0.799-0.974), with an accuracy of 0.880, a sensitivity of 0.850, a specificity of 0.900, a PPV of 0.850, and an NPV of 0.900. Comparing the three base models, the T2WI-based model best predicted axillary lymph node metastasis with an AUC of 0.908 (95% CI, 0.792-0.971). As a combination, the multiparametric MRI model was the best model for predicting axillary lymph node metastasis, and its AUC was 0.913 (95% CI, 0.799-0.974). The improvement compared with the AUC of the DWI base model and the DCE-MRI base model was significant (P< 0.01 for both). However, the increase was insignificant compared with the T2WI-based model (P=0.917).

**Table 3:** Performance characteristics of the base and multiparametric MRI models.

Model	ACC	SEN	SPE	PPV	NPV	AUC (95%CI)
T2WI	0.840	0.750	0.900	0.833	0.844	0.908 (0.792-0.971)
DWI	0.640	0.550	0.700	0.550	0.700	0.702 (0.556-0.823)
DCE-MRI	0.540	0.550	0.533	0.440	0.640	0.572 (0.424-0.711)
Multiparametric MRI	0.880	0.850	0.900	0.850	0.900	0.913 (0.799-0.974)

T2WI, T2-weighted imaging; DWI, diffusion weighting imaging; DCE-MRI, dynamic contrast-enhanced magnetic resonance imaging; ACC, accuracy; SEN, sensitivity; SPE, specificity; PPV, positive predictive value; NPV, negative predictive value; AUC, area under the receiver operating characteristic curve; CI, confidence interval.

## Discussion

This study developed a multiparametric MRI model incorporating ensemble learning to explore the performance of predicting axillary lymph node metastasis in invasive breast cancer before operation. To fully utilize the information from breast MRI, T2WI, DWI, and DCE-MRI were independent inputs to CNN model, and the predictions of the three base models were weighted and fused by ensemble learning based on weighted voting to achieve the final classification of axillary lymph nodes.

Deep learning is a method of feature learning in machine learning. It can automatically learn deep abstract features in the input data by simulating the mechanism of the human brain neural network processing information, reducing the dependence of human factors on crucial features and achieving an end-to-end learning effect, thus improving the extension ability of the model. Deep learning has been widely used in breast imaging, including segmentation (31, 32), differentiation of benign and malignant tumors (33, 34), and preoperative prediction related to breast cancer (35, 36).

There are only five studies on the preoperative evaluation of axillary lymph node status in breast cancer. Ha, et al. (24)and Ren et al. (25) studied only T1WI images of axillary lymph nodes using CNN models, and the accuracy of the CNN models in diagnosing axillary lymph node metastasis reached 0.843 and 0.848, respectively. Ren et al. (37) further analyzed T1WI, T2WI, and DCE-MRI of axillary lymph nodes, and the accuracy and AUC values of the three fused CNN models for assessing the status of axillary lymph nodes reached 0.885 and 0.882, respectively. Wang et al. (27)also investigated T1WI, T2WI, and DWI images, and their traditional CNN+SVM model was used for the deep-learning analysis of breast cancer tumors. The T1WI+T2WI+DWI model had the best diagnostic efficacy for axillary lymph node metastasis, with accuracy and AUC values of 0.970 and 0.996. Luo et al. (26)developed a CNN+SVM model to extract deep features from DWI images of different orientations of the breast cancer tumor by using the CNN model and SVM as a classifier. They then evaluated the status of axillary lymph nodes, and the AUC value of this model was 0.852. These studies suggest that deep learning analysis of MRI images of the breast cancer tumor and axillary lymph nodes would be useful for predicting axillary lymph node status in breast cancer patients.

In this study, axial T2WI, DWI, and DCE-MRI images of breast cancer tumors of the same resolution size were used for deep learning analysis to ensure consistency of image input. The efficacy of the T2WI-based model for diagnosing axillary lymph node metastasis remained superior to that of the DWI and DCE-MRI-based models. The results were consistent with those of Wang et al (27). The possible reasons are mainly in two aspects. First, the T2WI-based model may extract richer features than the DWI and DCE-MRI base models in our study. ResNets are widely used in image classification tasks, such like ResNet18, ResNet50, ResNet101, but the number of extracted features varies with the number of layers of the ResNet. The feature extraction process is generally simple to complex and low-level to high-level. The ResNet50 model may be too shallow for DWI and DCE-MRI images in terms of the number of layers to extract relevant deep features. Second, for axillary lymph node metastasis, T2WI raw images of invasive breast cancer have more meaningful features than DWI and DCE-MRI raw images. The ROI in this study included the breast tumor itself and peritumoral edema. Previous studies (38)showed that the presence of perineural edema in breast cancer and axillary lymph node metastasis were closely related, and T2WI was the best sequence to show perineural edema.

Our study has some limitations. First, this study is a retrospective analysis and lacks multicenter data, which needs to be further validated by prospective studies. Second, the sample size is small, and the data distribution is unbalanced between ALNM and Non-ALNM groups, which needs a more extensive data set for further study. Third, the MR images acquired in this study are from two types of MR scanners, which may have some influence on the accuracy of the final results. However, based on the idea of multicenter validation, we believe that the model in our study could achieve similar accuracy in different medical centers and has the value of clinical replication.

In conclusion, we developed a novel model for preoperative prediction of axillary lymph node metastasis in breast cancer using multi-parametric MRI datasets. Our results showed that T2WI outperformed DWI and DCE-MRI for predicting axillary lymph node metastasis in breast cancer for a single MRI sequence, but the multiparametric MRI model combined with ensemble learning improved the predictive performance. This model may give clinicians more information about breast cancer axillary lymph nodes preoperatively and assist in clinical decision-making.

## Data availability statement

The original contributions presented in the study are included in the article/supplementary material. Further inquiries can be directed to the corresponding author.

## Ethics statement

The studies involving human participants were reviewed and approved by the ethics committee and institutional review of The First Affiliated Hospital of Shandong First Medical University. The ethics committee waived the requirement of written informed consent for participation. Written informed consent was not obtained from the individual(s) for the publication of any potentially identifiable images or data included in this article.

## Author contributions

XZ: Data investigation, experiment design, manuscript writing; ML: Experiment guidance and statistical method selection; WR and JS: Data collection and analysis; KW and XX: Data processing and analysis; GZ: Experiment Design, Supervision, Writing – Review. All authors contributed to the article and approved the submitted version.

## References

[1] Sung H Ferlay J Siegel RL Laversanne M Soerjomataram I Jemal A. Global cancer statistics 2020: Globocan estimates of incidence and mortality worldwide for 36 cancers in 185 countries. CA Cancer J Clin (2021) 71(3):209–49. doi: 10.3322/caac.21660 33538338

[2] Chang JM Leung JWT Moy L Ha SM Moon WK. Axillary nodal evaluation in breast cancer: State of the art. Radiology (2020) 295(3):500–15. doi: 10.1148/radiol.2020192534 32315268

[3] Maxwell F de Margerie Mellon C Bricout M Cauderlier E Chapelier M Albiter M. Diagnostic strategy for the assessment of axillary lymph node status in breast cancer. Diagn Interv Imaging (2015) 96(10):1089–101. doi: 10.1016/j.diii.2015.07.007 26372221

[4] Rahman M Mohammed S. Breast cancer metastasis and the lymphatic system. Oncol Lett (2015) 10(3):1233–9. doi: 10.3892/ol.2015.3486 PMC4533217 26622656

[5] Duff M Hill AD McGreal G Walsh S McDermott EW O’Higgins NJ. Prospective evaluation of the morbidity of axillary clearance for breast cancer. Br J Surg (2001) 88(1):114–7. doi: 10.1046/j.1365-2168.2001.01620.x 11136322

[6] Langer I Guller U Berclaz G Koechli OR Schaer G Fehr MK. Morbidity of sentinel lymph node biopsy (Sln) alone versus sln and completion axillary lymph node dissection after breast cancer surgery: A prospective Swiss multicenter study on 659 patients. Ann Surg (2007) 245(3):452–61. doi: 10.1097/01.sla.0000245472.47748.ec PMC1877006 17435553

[7] Kootstra JJ Dijkstra PU Rietman H de Vries J Baas P Geertzen JH. A longitudinal study of shoulder and arm morbidity in breast cancer survivors 7 years after sentinel lymph node biopsy or axillary lymph node dissection. Breast Cancer Res Treat (2013) 139(1):125–34. doi: 10.1007/s10549-013-2509-y 23588950

[8] Ahmed M Usiskin SI Hall-Craggs MA Douek M. Is imaging the future of axillary staging in breast cancer? Eur Radiol (2014) 24(2):288–93. doi: 10.1007/s00330-013-3009-5 24037250

[9] Hwang SO Lee SW Kim HJ Kim WW Park HY Jung JH. The comparative study of ultrasonography, contrast-enhanced mri, and (18)F-fdg Pet/Ct for detecting axillary lymph node metastasis in T1 breast cancer. J Breast Cancer (2013) 16(3):315–21. doi: 10.4048/jbc.2013.16.3.315 PMC3800728 24155761

[10] Shetty MK Carpenter WS. Sonographic evaluation of isolated abnormal axillary lymph nodes identified on mammograms. J Ultrasound Med (2004) 23(1):63–71. doi: 10.7863/jum.2004.23.1.63 14756355

[11] Alvarez S Añorbe E Alcorta P López F Alonso I Cortés J. Role of sonography in the diagnosis of axillary lymph node metastases in breast cancer: A systematic review. AJR Am J Roentgenol (2006) 186(5):1342–8. doi: 10.2214/ajr.05.0936 16632729

[12] Zhang X Liu Y Luo H Zhang J. Pet/Ct and mri for identifying axillary lymph node metastases in breast cancer patients: Systematic review and meta-analysis. J Magn Reson Imaging (2020) 52(6):1840–51. doi: 10.1002/jmri.27246 32567090

[13] Liang X Yu J Wen B Xie J Cai Q Yang Q. Mri and fdg-Pet/Ct based assessment of axillary lymph node metastasis in early breast cancer: A meta-analysis. Clin Radiol (2017) 72(4):295–301. doi: 10.1016/j.crad.2016.12.001 28139203

[14] Yoshimura G Sakurai T Oura S Suzuma T Tamaki T Umemura T. Evaluation of axillary lymph node status in breast cancer with mri. Breast Cancer (1999) 6(3):249–58. doi: 10.1007/bf02967179 11091725

[15] Mortellaro VE Marshall J Singer L Hochwald SN Chang M Copeland EM. Magnetic resonance imaging for axillary staging in patients with breast cancer. J Magn Reson Imaging (2009) 30(2):309–12. doi: 10.1002/jmri.21802 19466713

[16] Schipper RJ Paiman ML Beets-Tan RG Nelemans PJ de Vries B Heuts EM. Diagnostic performance of dedicated axillary T2- and diffusion-weighted Mr imaging for nodal staging in breast cancer. Radiology (2015) 275(2):345–55. doi: 10.1148/radiol.14141167 25513854

[17] Choi SH Moon WK. Contrast-enhanced Mr imaging of lymph nodes in cancer patients. Kor J Radiol (2010) 11(4):383–94. doi: 10.3348/kjr.2010.11.4.383 PMC2893309 20592922

[18] Zhou P Wei Y Chen G Guo L Yan D Wang Y. Axillary lymph node metastasis detection by magnetic resonance imaging in patients with breast cancer: A meta-analysis. Thorac Cancer (2018) 9(8):989–96. doi: 10.1111/1759-7714.12774 PMC6068453 29877048

[19] Zhao M Wu Q Guo L Zhou L Fu K. Magnetic resonance imaging features for predicting axillary lymph node metastasis in patients with breast cancer. Eur J Radiol (2020) 129:109093. doi: 10.1016/j.ejrad.2020.109093 32512504

[20] Razek AA Lattif MA Denewer A Farouk O Nada N. Assessment of axillary lymph nodes in patients with breast cancer with diffusion-weighted Mr imaging in combination with routine and dynamic contrast Mr imaging. Breast Cancer (2016) 23(3):525–32. doi: 10.1007/s12282-015-0598-7 25763535

[21] Dong Y Feng Q Yang W Lu Z Deng C Zhang L. Preoperative prediction of sentinel lymph node metastasis in breast cancer based on radiomics of T2-weighted fat-suppression and diffusion-weighted mri. Eur Radiol (2018) 28(2):582–91. doi: 10.1007/s00330-017-5005-7 28828635

[22] Han L Zhu Y Liu Z Yu T He C Jiang W. Radiomic nomogram for prediction of axillary lymph node metastasis in breast cancer. Eur Radiol (2019) 29(7):3820–9. doi: 10.1007/s00330-018-5981-2 30701328

[23] LeCun Y Bengio Y Hinton G. Deep learning. Nature (2015) 521(7553):436–44. doi: 10.1038/nature14539 26017442

[24] Ha R Chang P Karcich J Mutasa S Fardanesh R Wynn RT. Axillary lymph node evaluation utilizing convolutional neural networks using mri dataset. J Digit Imaging (2018) 31(6):851–6. doi: 10.1007/s10278-018-0086-7 PMC6261196 29696472

[25] Ren T Cattell R Duanmu H Huang P Li H Vanguri R. Convolutional neural network detection of axillary lymph node metastasis using standard clinical breast mri. Clin Breast Cancer (2020) 20(3):e301–e8. doi: 10.1016/j.clbc.2019.11.009 32139272

[26] Luo J Ning Z Zhang S Feng Q Zhang Y. Bag of deep features for preoperative prediction of sentinel lymph node metastasis in breast cancer. Phys Med Biol (2018) 63(24):245014. doi: 10.1088/1361-6560/aaf241 30523819

[27] Wang Z Sun H Li J Chen J Meng F Li H. Preoperative prediction of axillary lymph node metastasis in breast cancer using cnn based on multiparametric mri. J Magn Reson Imaging (2022) 56(3):700–9. doi: 10.1002/jmri.28082 35108415

[28] Sun R Meng Z Hou X Chen Y Yang Y Huang G. Prediction of breast cancer molecular subtypes using dce-mri based on cnns combined with ensemble learning. Phys Med Biol (2021) 66(17). doi: 10.1088/1361-6560/ac195a 34330117

[29] Yoshihara E Smeets A Laenen A Reynders A Soens J Van Ongeval C. Predictors of axillary lymph node metastases in early breast cancer and their applicability in clinical practice. Breast (2013) 22(3):357–61. doi: 10.1016/j.breast.2012.09.003 23022046

[30] Kim JY Seo HB Park S Moon JI Lee JW Lee NK. Early-stage invasive ductal carcinoma: Association of tumor apparent diffusion coefficient values with axillary lymph node metastasis. Eur J Radiol (2015) 84(11):2137–43. doi: 10.1016/j.ejrad.2015.08.009 26318821

[31] Ha R Chang P Mema E Mutasa S Karcich J Wynn RT. Fully automated convolutional neural network method for quantification of breast mri fibroglandular tissue and background parenchymal enhancement. J Digit Imaging (2019) 32(1):141–7. doi: 10.1007/s10278-018-0114-7 PMC6382627 30076489

[32] Piantadosi G Sansone M Fusco R Sansone C. Multi-planar 3d breast segmentation in mri *Via* deep convolutional neural networks. Artif Intell Med (2020) 103:101781. doi: 10.1016/j.artmed.2019.101781 32143788

[33] Adachi M Fujioka T Mori M Kubota K Kikuchi Y Xiaotong W. Detection and diagnosis of breast cancer using artificial intelligence based assessment of maximum intensity projection dynamic contrast-enhanced magnetic resonance images. Diag (Basel) (2020) 10(5). doi: 10.3390/diagnostics10050330 PMC7277981 32443922

[34] Hu Q Whitney HM Giger ML. A deep learning methodology for improved breast cancer diagnosis using multiparametric mri. Sci Rep (2020) 10(1):10536. doi: 10.1038/s41598-020-67441-4 32601367 PMC7324398

[35] Sun R Hou X Li X Xie Y Nie S. Transfer learning strategy based on unsupervised learning and ensemble learning for breast cancer molecular subtype prediction using dynamic contrast-enhanced mri. J Magn Reson Imaging (2022) 55(5):1518–34. doi: 10.1002/jmri.27955 34668601

[36] Qu YH Zhu HT Cao K Li XT Ye M Sun YS. Prediction of pathological complete response to neoadjuvant chemotherapy in breast cancer using a deep learning (Dl) method. Thorac Cancer (2020) 11(3):651–8. doi: 10.1111/1759-7714.13309 PMC7049483 31944571

[37] Ren T Lin S Huang P Duong TQ. Convolutional neural network of multiparametric mri accurately detects axillary lymph node metastasis in breast cancer patients with pre neoadjuvant chemotherapy. Clin Breast Cancer (2022) 22(2):170–7. doi: 10.1016/j.clbc.2021.07.002 34384696

[38] Liang T Hu B Du H Zhang Y. Predictive value of T2-weighted magnetic resonance imaging for the prognosis of patients with mass-type breast cancer with peritumoral edema. Oncol Lett (2020) 20(6):314. doi: 10.3892/ol.2020.12177 33133250 PMC7590426

